# Emergence of Non-albicans Candida Species in Candiduria Among Diabetic Patients: A Cross-Sectional Study

**DOI:** 10.7759/cureus.108577

**Published:** 2026-05-10

**Authors:** Vaishnavi D Patil, Priyanka M Mane, Satish R Patil

**Affiliations:** 1 Department of Microbiology, Krishna Institute of Medical Sciences, Krishna Vishwa Vidyapeeth (Deemed to be University), Karad, IND

**Keywords:** candida albicans, candiduria, chromagar candida, diabetes mellitus, non-albicans candida

## Abstract

Background and purpose: Fungal urinary tract infections are increasingly recognized in clinical practice, particularly among patients with diabetes mellitus. These infections may pose significant health risks due to underlying immune dysfunction. This study aimed to determine the distribution of *Candida *species in candiduria among patients with type 2 diabetes mellitus using conventional and CHROMagar *Candida *methods.

Materials and methods: Patients with diabetes mellitus who were referred to Krishna Hospital in Karad, India, participated in this cross-sectional, descriptive study. To determine the causative agents, urine specimens were obtained and cultured. Culture-positive samples were examined using standard identification tests and colony color on CHROMagar *Candida *medium.

Results: *Candida albicans* was the most prevalent isolate among the 85 samples examined in this study (n=34; 40%), followed by *C. krusei* (n=20; 23.53%), *C. tropicalis* (n=17; 20%), and *C. glabrata* (n=14; 16.47%).

Conclusion: Non-albicans *Candida *species predominated among candiduria isolates in diabetic patients, highlighting the need for accurate species identification to guide appropriate antifungal management.

## Introduction

Despite being commensal organisms of mucosal surfaces, *Candida* species can cause illness when they penetrate organs due to weakened mucosal barriers or damaged local or systemic immune systems [1]. Urinary tract infections (UTIs) are among the most prevalent infections in clinical practice and are caused by a variety of pathogens, including bacteria, fungi, viruses, and parasites [2-4]. *Candida *species are the most prevalent organisms causing fungal UTIs. Despite its rare occurrence in the community, *Candida *UTIs are primarily observed in hospitalized patients [5].

Pregnancy, long-term antibiotic use, diabetes mellitus, corticosteroid use, human immunodeficiency virus (HIV) infection, and immunocompromised conditions significantly contribute to disease susceptibility [6]. Candiduria can indicate recurrent or disseminated candidiasis, fungal bezoars, and bladder colonization from indwelling catheters [7]. However, diabetes is one of the main risk factors for developing it [8,9]. The clinical significance of candiduria is highlighted by the elevated risk of mortality and morbidity in immunocompromised individuals [10].

Diabetes mellitus is a major predisposing factor for candiduria due to multiple underlying pathophysiological mechanisms. Persistent hyperglycemia impairs host immune responses, including neutrophil function and cellular immunity, thereby reducing the ability to control fungal proliferation [9]. In addition, glycosuria creates a nutrient-rich environment that promotes the growth and colonization of *Candida *species within the urinary tract [8]. Diabetic patients are therefore at a higher risk of developing UTIs, including those caused by *Candida *species, compared to non-diabetic individuals [6,9]. The clinical significance of candiduria in such patients is further emphasized by its association with increased morbidity and the potential for progression to invasive infections, particularly in immunocompromised states [7,10]. This study aimed to determine the distribution of *Candida *species in candiduria among patients with type 2 diabetes mellitus using conventional and CHROMagar *Candida *methods.

## Materials and methods

This was a cross-sectional descriptive study carried out at Krishna Charitable Hospital and Medical Research Centre, Karad, India. The ethical clearance for the study was obtained from the Institutional Ethics Committee of Krishna Vishwa Vidyapeeth (Deemed to be University) (approval number: KVV/IEC/05/2024).

Inclusion and exclusion criteria

Patients diagnosed with type 2 diabetes mellitus and clinically suspected of having a UTI were included in the study. Non-diabetic individuals and patients with type 1 diabetes mellitus were excluded. A total of 85 urine samples were collected from eligible type 2 diabetic patients with suspected UTI during the study period.

Sample collection and processing

Midstream clean-catch urine samples were collected in sterile, labelled universal containers following standard aseptic precautions. The samples were transported promptly to the microbiology laboratory and processed without delay. The specimen was inoculated onto Sabouraud dextrose agar (SDA) supplemented with chloramphenicol and incubated at 35°C for 48-72 hours. The resulting colonies were examined for growth characteristics suggestive of *Candida *species [11].

Microscopic Examination

A few colonies from a pure culture were placed onto a clean glass slide to create a smear, which was then subjected to Gram staining. Gram staining revealed Gram-positive budding yeast cells with pseudohyphae [11].

Identification of Candida Species

Further identification was performed using the germ tube test; a few colonies from pure culture were suspended in 0.5 ml of pooled serum in a test tube and incubated at 37°C for 2-3 hours. After incubation, a drop of the suspension was placed on a glass slide, covered with a coverslip, and examined microscopically for germ tube formation [12].

For morphological characterization, *Candida *isolates were grown on cornmeal agar (CMA) and incubated at 25°C. After 2-3 days of incubation, morphological features such as pseudohyphae and chlamydospore formation were observed microscopically, which aided in species identification [11].

Sugar Fermentation Test

Carbohydrate fermentation tests were performed to differentiate *Candida *species based on their ability to ferment sugars. Glucose, sucrose, lactose, and maltose were used as substrates. Acid production was indicated by a change in the color of the pH indicator, while gas production was detected by the presence of bubbles in the Durham tube.

CHROMagar Candida Medium

Additionally, isolates obtained from purity plates were streaked onto CHROMagar *Candida *medium using a sterile inoculating loop and incubated at 37°C for 48 hours. This technique relies on the differential appearance of colony color characteristics of various *Candida *species [13]. Yeast identification was performed based on colony color and morphological traits [14].

Standard laboratory quality control procedures were followed throughout the culture and identification processes to ensure the accuracy and reliability of results.

Statistical analysis

This was a descriptive cross-sectional study; only descriptive statistics (frequency and percentage) were used for data analysis, and no inferential statistical tests were applied.

## Results

A total of 85 urine samples from patients with type 2 diabetes mellitus were analyzed in this study. All samples demonstrated significant candiduria, defined as a colony count ≥10³ CFU/mL.

Among the 85 cases studied, 47 (55.3%) were male, and 38 (44.7%) were female. The majority of cases were observed in the age group of 61-70 years (42.35%), followed by 41-60 years (29.41%), 71-90 years (23.53%), and 20-40 years (4.71%) (Table 1).

**Table 1:** Age- and gender-wise patient distribution of isolated Candida species n: number; %: percentage

Age group (years)	Gender		Total %
	Male (n=47)	Female (n=38)	
20-40	1	3	4 (4.71)
41-60	14	11	25 (29.41)
61-70	17	19	36 (42.35)
71-90	15	5	20 (23.53)

Microscopic findings

**Figure 1:**
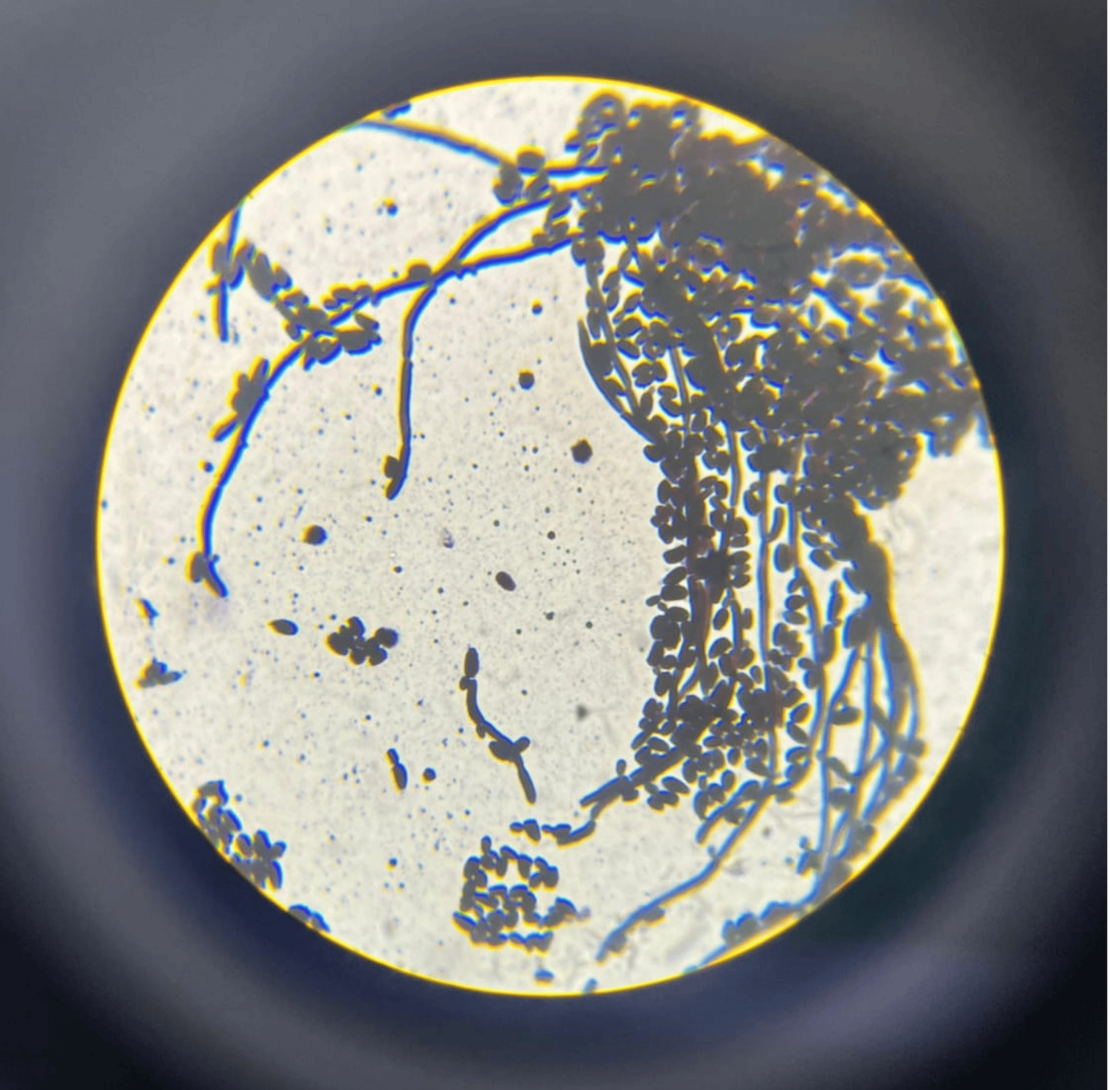
Gram stain of Candida (Gram staining ×100)

Gram staining of the isolates revealed Gram-positive budding yeast cells with pseudohyphae, consistent with *Candida *species (Figure 1).

**Figure 2:**
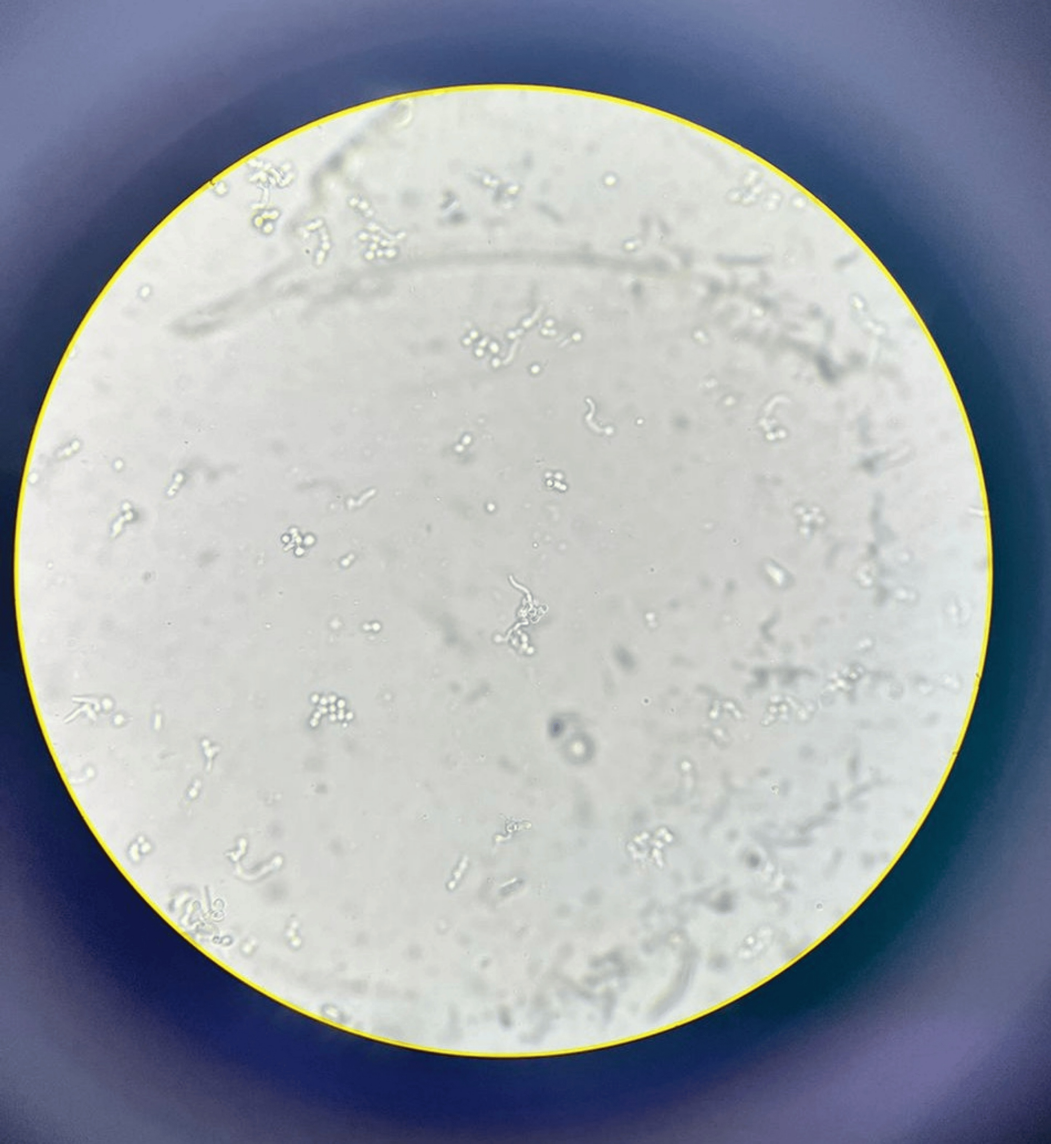
Germ tube seen in Candida albicans (serum ×40)

The germ tube test demonstrated germ tube formation in isolates identified as *C. albicans*, confirming its presence (Figure 2).

Species identification by conventional methods

Morphological examination on cornmeal agar showed characteristic features such as pseudohyphae and chlamydospore formation, aiding in species identification. Sugar fermentation tests further differentiated *Candida *species based on their carbohydrate utilization patterns.

All *Candida *species fermented glucose, whereas sucrose and lactose fermentation were observed only in *C. tropicalis*. Maltose fermentation was observed in *C. albicans*, whereas *C. krusei *and *C. glabrata* fermented only glucose. Based on sugar fermentation patterns, 34 (40%) of the isolates were recognized as *C. albicans*, followed by *C. krusei* (20, 23.53%), *C. tropicalis* (17, 20%), and *C. glabrata* (14, 16.47%), as displayed in Table 2.

**Table 2:** Distribution of Candida species according to sugar fermentation tests The table shows the number (n) and percentage of different *Candida *species identified based on their carbohydrate fermentation profiles. Each species demonstrates a characteristic pattern of sugar utilization, which aids in differentiation and identification. G: glucose; S: sucrose; L: lactose; M: maltose; AG: acid and gas production; -: no fermentation (negative reaction); %: percentage

G	S	L	M	*Candida *species identified (n)	Total %
AG	-	-	AG	*C. albicans* (34)	40
AG	-	-	-	*C. krusei* (20)	23.53
AG	AG	AG	-	*C. tropicalis* (17)	20
AG	-	-	-	*C. glabrata* (14)	16.47

**Figure 3:**
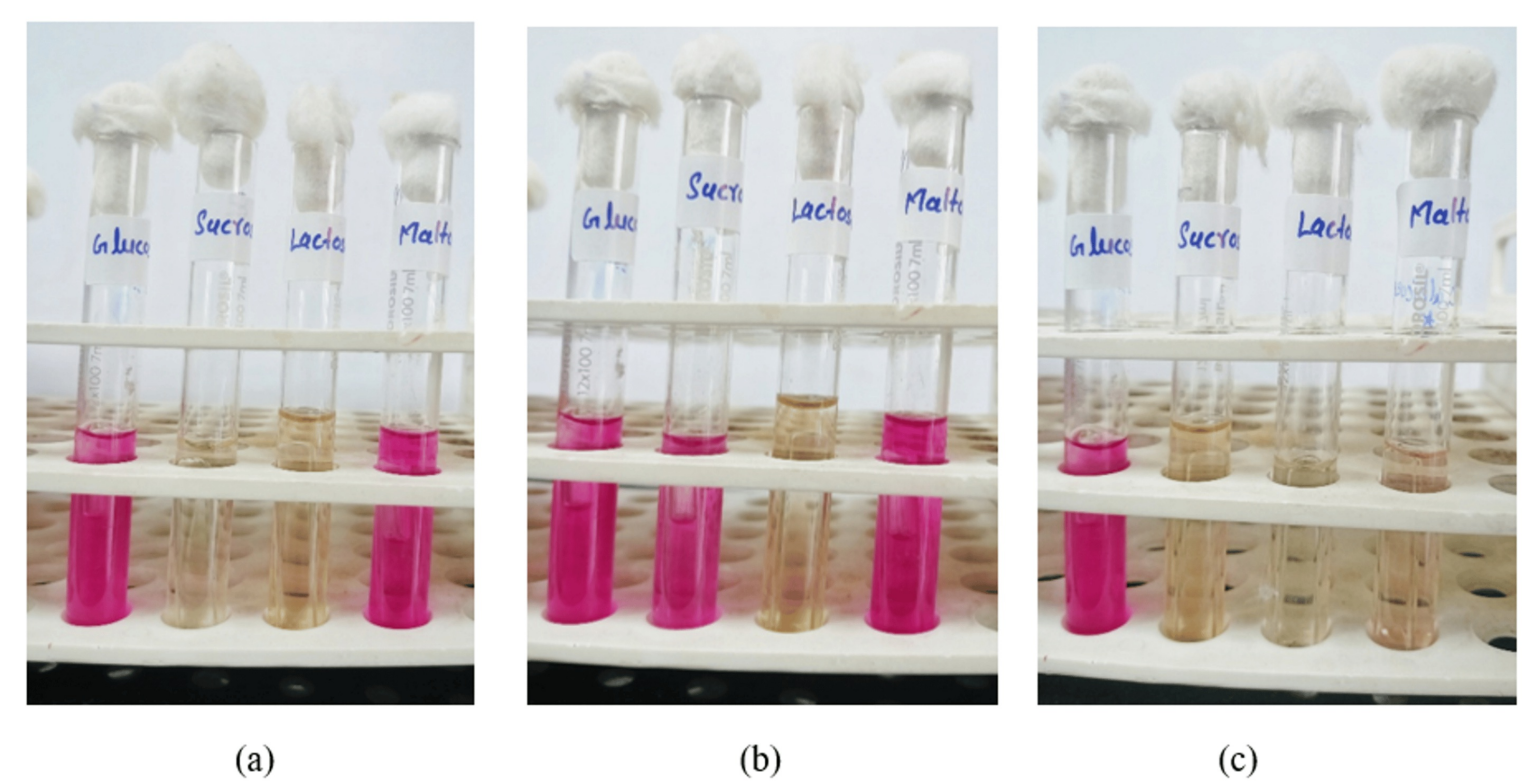
Sugar fermentation reaction by different species of Candida: (a) Candida albicans, (b) Candida tropicalis, and (c) Candida krusei and Candida glabrata

The sugar fermentation reactions of different *Candida *species are illustrated in Figure 3.

Species identification by CHROMagar *Candida*

**Figure 4:**
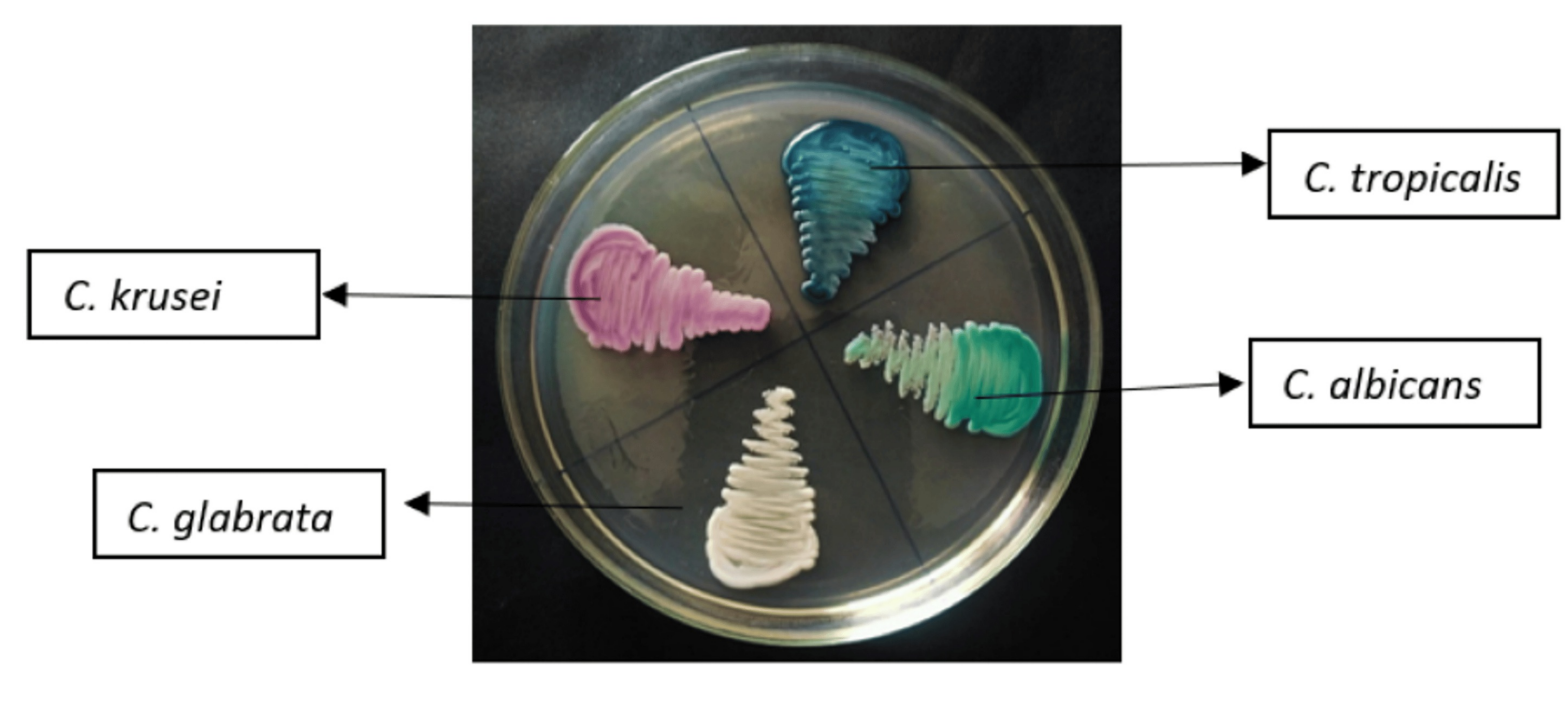
CHROMagar Candida displaying the color-coded growth of several Candida species

On CHROMagar *Candida *medium, isolates exhibited distinct colony colors that facilitated rapid identification. *C. albicans* produced green colonies, *C. tropicalis* showed metallic blue colonies, *C. krusei* appeared as purple colonies, and *C. glabrata *produced white- to cream-colored colonies (Figure 4).

The distribution of *Candida *species based on colony color is presented in Table 3.

**Table 3:** Distribution of Candida species according to colony color on CHROMagar The table shows the *Candida *species distribution on CHROMagar *Candida *medium according to colony color. On CHROMagar, distinctive colony colors are produced by various *Candida *species, which helps with presumptive identification. *Candida albicans *is represented by green colonies, *Candida krusei *by purple colonies, *Candida tropicalis* by metallic blue colonies, and *Candida glabrata* by white to cream colonies. For each species, the number of isolates (no.) is displayed.

Colony color on CHROMagar	*Candida *spp. identified (no.)
Green	*C. albicans* (34)
Purple	*C. krusei *(20)
Metallic blue	*C. tropicalis* (17)
White to cream	*C. glabrata* (14)

Comparison of the results obtained from sugar fermentation tests and CHROMagar *Candida *methods revealed that both approaches gave comparable identification outcomes for *C. albicans, C. krusei, C. tropicalis, *and *C. glabrata.* However, sugar fermentation alone showed overlapping results for certain species, whereas differentiation based on colony color on CHROMagar *Candida *provided clearer species identification.

## Discussion

In the present study, 85 diabetic patients with candiduria were evaluated, and a predominance of non-albicans *Candida *species (60%) over *C. albicans* (40%) was observed. This finding suggests a shifting trend in the epidemiology of *Candida *infections, particularly among patients with diabetes mellitus, who are at increased risk due to underlying immunological alterations.

The predominance of non-albicans *Candida *species in this study is consistent with current global trends, where an increasing incidence of non-albicans *Candida *has been reported in clinical infections [15]. However, some studies have reported a predominance of *C. albicans*, which contrasts with the present findings. Diabetes mellitus is a well-established risk factor for UTIs, including candiduria, and several studies have highlighted its role in predisposing individuals to such infections [16]. Variations in prevalence rates reported across studies may be attributed to differences in study populations, underlying risk factors, healthcare practices, and geographical distribution [17-19].

Among the non-albicans *Candida *isolates identified in this study, *C. krusei *(23.53%) and *C. tropicalis* (20%) were the most common species, followed by *C. glabrata* (16.47%). These findings are comparable with those reported by Ali et al., who also observed a similar distribution of *Candida *species in clinical isolates [20]. The increasing isolation of these species is clinically significant, as they are often associated with reduced susceptibility to commonly used azole antifungal agents. This reduced susceptibility may contribute to therapeutic challenges and highlights the importance of accurate species identification in guiding appropriate antifungal therapy [21,22].

Conventional methods such as germ tube testing, chlamydospore formation on cornmeal agar, and sugar fermentation tests were utilized for species identification in the present study. While these methods are widely used, they are labor-intensive and time-consuming and may yield overlapping results, particularly in carbohydrate fermentation profiles. In contrast, CHROMagar *Candida *medium offers a rapid and reliable method for presumptive identification based on colony color differentiation. In this study, CHROMagar demonstrated high concordance with conventional methods while providing faster results, thereby facilitating early diagnosis and management.

The findings of this study underscore the importance of timely and accurate identification of *Candida *species in diabetic patients with candiduria. The increasing prevalence of non-albicans *Candida *species necessitates routine species-level identification, as it has direct implications for antifungal therapy and patient outcomes.

However, certain limitations of the study must be acknowledged. It was not possible to differentiate between colonization and true infection, which may affect the clinical interpretation of candiduria. Additionally, antifungal susceptibility testing was not performed, limiting the ability to assess resistance patterns among the isolates. Molecular identification of *Candida *species was not performed, which may limit precise species confirmation. The relatively small sample size and lack of statistical analysis may also limit the generalizability of the findings. Further studies incorporating larger populations, antifungal susceptibility testing, and clinical correlation are recommended to better understand the clinical significance of candiduria in diabetic patients.

Despite these limitations, a key strength of the present study is the use of both conventional and chromogenic methods for species identification, allowing for a comparative evaluation of diagnostic approaches. The study also contributes valuable data regarding the distribution of *Candida *species in a high-risk population, thereby adding to the existing body of knowledge in this field.

## Conclusions

This study demonstrates that non-albicans *Candida *species predominate among candiduria isolates in patients with type 2 diabetes mellitus, accounting for 60% of cases, while *C. albicans* constitutes 40%. The increasing prevalence of non-albicans *Candida *species is clinically significant due to their reduced susceptibility to commonly used antifungal agents, emphasizing the need for accurate species-level identification. Conventional identification methods, although useful, are time-consuming and may yield overlapping results. In contrast, CHROMagar *Candida *medium provides a rapid and reliable method for the presumptive identification of *Candida *species, facilitating early diagnosis and appropriate management.

Early detection and targeted treatment of candiduria in diabetic patients are essential to reduce potential complications and improve clinical outcomes. Further studies incorporating antifungal susceptibility testing and larger sample sizes are recommended to enhance the understanding and management of candiduria in this high-risk population.
